# Dentin Bond Strength of Dental Adhesives Functionalized with Polyhedral Oligomeric Silsesquioxanes

**DOI:** 10.3390/ma17061321

**Published:** 2024-03-13

**Authors:** Jana Biermann, Charlyn Elise Bień, Clemens Lechte, Philipp Kanzow, Annette Wiegand

**Affiliations:** Department of Preventive Dentistry, Periodontology and Cariology, University Medical Center Göttingen, 37075 Göttingen, Germany; bien.charlyn@gmx.de (C.E.B.); clemens.lechte@med.uni-goettingen.de (C.L.); philipp.kanzow@med.uni-goettingen.de (P.K.); annette.wiegand@med.uni-goettingen.de (A.W.)

**Keywords:** bond strength, dental adhesive, polyhedral oligomeric silsesquioxanes, POSS, SBS, water storage

## Abstract

This study analyzed the dentin shear bond strength (SBS) of an etch-and-rinse (ER) or a self-etch (SE) adhesive incorporated with multifunctional polyhedral oligomeric silsesquioxanes (MA-POSS-8). An ER adhesive (Solobond Plus, VOCO GmbH, Cuxhaven, Germany) and a universal adhesive applied in SE mode (Scotchbond Universal, 3M, St. Paul, MN, USA) were infiltrated with MA-POSS-8 (Hybrid Plastics Inc., Hattiesburg, MS, USA) at 5 wt.% or 10 wt.%. Pure adhesives served as controls. Bovine dentin specimens were conditioned with one of the adhesives prior to the application of a nano-hybrid composite (Venus Diamond A3, Kulzer, Hanau, Germany). SBS and failure modes were determined after water storage for 24 h, 6 months, 12 months, or 24 months (each subgroup n = 20). Statistical analysis was performed using ANOVAs, Weibull statistics, and χ^2^ tests (*p* < 0.05). SBSs for the control groups after 24 h were 17.4 ± 4.9 MPa for the ER adhesive and 19.1 ± 5.2 MPa for the universal adhesive. After 24 months, the SBS of the ER adhesive was significantly higher for 5 wt.% MA-POSS-8 (17.9 ± 5.1 MPa) than for the control group (14.6 ± 3.6 MPa) and 10 wt.% MA-POSS-8 (12.8 ± 4.1 MPa), and more cohesive failures were observed. The SBS of the universal adhesive increased during aging, irrespective of the MA-POSS-8 concentration. 5 wt.% MA-POSS-8 improves the SBS of the ER adhesive and does not impair the SBS of the SE adhesive.

## 1. Introduction

The bond strength of dental adhesives on dentin substantively depends on the stability of the hybrid layer between both materials [1]. The hybrid layer constitutes a compound between the collagen fibers of the dentin and the monomers of the applied dental adhesive, thus enabling a bond between a hydrophilic and a hydrophobic substance [2]. Unfortunately, the hydrolytic and enzymatic degradation of exposed collagen fibrils and hydrophilic resin components over time leads to a disintegration of the hybrid layer, resulting in a less stable bond between dentin and dental adhesive and consequently the adhesive restoration [1]. Therefore, combating the degradation of the hybrid layer has become of major interest. One concept is to enhance the adhesive with functionalized nanoparticles that ameliorate its mechanical and biological properties [3,4,5]. The incorporation of polyhedral oligomeric silsesquioxanes (POSSs) into dental adhesives seems to be a promising approach towards that direction, as they evolve bioactive potential by the stimulation of mineral precipitation on the surface to support the stability of the hybrid layer [6].

POSSs are nanostructured organic–inorganic hybrid molecules. Their inorganic core consists of silicon and oxygen and is surrounded by organic groups. In multifunctional POSSs, several of these organic groups are reactive and thus allow polymerization [7]. The hybrid character of POSSs leads to good dispersion and particle mobility when incorporated into organic substances [8] and their multifunctionality results in a high cross-linking density [9]. The experimental incorporation of POSSs into composites improved their physical properties such as hardness and flexural strength and decreased the volumetric shrinkage [10]. POSS-containing materials indicated mineralizing capacity by promoting the formation of hydroxyapatite crystals on their surfaces in vitro [11,12].

A previous study asserted that dental adhesives modified with multifunctional POSSs present bioactive potential by inducing the formation of calcium phosphate precipitates without crucially compromised material properties [6]. However, the principal task of an adhesive system is to enable efficient and long-term bonding to the tooth structure. Thus, analyzing the bond strength of adhesive systems incorporated with MA-POSS-8 is a crucial step towards their clinical applicability. The immediate bond strength of experimental adhesives seemed to improve through the incorporation of POSSs [4,13], but the effects of POSSs on the bond strength of commercially available dental adhesives, especially after aging, are yet unknown.

Therefore, the aim of the present study was to analyze the dentin bond strength of two different dental adhesives (an etch-and-rinse adhesive and a universal adhesive in self-etch mode) that were incorporated with multifunctional POSSs. The null hypothesis was that the dentin shear bond strength of the functionalized adhesives does not differ significantly from the dentin bond strength of the neat adhesives.

## 2. Materials and Methods

### 2.1. Adhesive Modification

**Figure 1:**
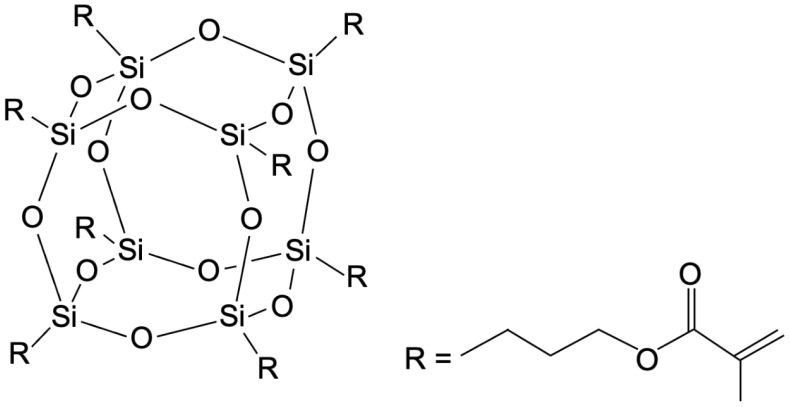
Chemical structure of MA-POSS-8. MA-POSS-8 is a nanostructured organic–inorganic hybrid molecule. The inorganic core consists of silicon and oxygen and is surrounded by eight reactive groups that allow polymerization.

An etch-and-rinse (ER) adhesive (Solobond Plus, VOCO GmbH, Cuxhaven, Germany) and a universal adhesive in self-etch (SE) mode (Scotchbond Universal, 3M, St. Paul, MN, USA) were incorporated with multifunctional POSSs (Hybrid Plastics Inc., Hattiesburg, MS, USA). For the modification of both adhesive systems, a methacryl-functionalized POSS bearing eight reactive groups per particle (MA-POSS-8, Figure 1) was used. MA-POSS-8 was added in two different concentrations (5 wt.% and 10 wt.%) to each adhesive. For the ER adhesive, MA-POSS-8 was incorporated into the adhesive, while the primer was not modified. As the universal adhesive was a single-bottle adhesive, the entire adhesive system was infiltrated with MA-POSS-8. The detailed compositions of both adhesive systems are listed in Table 1. After MA-POSS-8 was added, the mixtures were magnetically stirred in the dark for 5 min, following a previously applied protocol verifying a good dispersion of MA-POSS-8 particles in acetone- and ethanol-based adhesives [6,14]. The modified adhesives were kept in a lightproof container and were further processed immediately after preparation. For the control groups, both adhesives were left unchanged.

### 2.2. Specimen Preparation

Dentin specimens (n = 480) were obtained from bovine permanent teeth that were taken from slaughterhouses as waste products in the slaughter process. The crowns were embedded in resin and ground under water cooling (silicon carbide paper P500, Hermes Schleifmittel GmbH, Hamburg, Germany; cutting wheel Roto-Pol34, Struers GmbH, Willich, Germany) until a flat dentin surface emerged. Half of the specimens were then treated with the ER adhesive, and the other half with the universal adhesive, following the manufacturers’ instructions as follows:

For the ER adhesive, the dentin surface was etched for 15 s (Ultra-Etch, Ultradent Products Inc., South Jordan, UT, USA) and thoroughly rinsed with water for 20 s. The surplus water was removed but the surface was not completely dried. The primer was distributed for 30 s and airdried. The adhesive was applied for 15 s, gently dispersed with air, and light-cured (BA Optima 10, B.A. international, Northampton, UK, output > 800 mW/cm^2^) for 20 s.

The universal adhesive was applied in SE mode. It was distributed for 20 s on the dentin surface and gently airdried for 5 s. The adhesive was light-cured (BA Optima 10, B.A. international, UK, output > 800 mW/cm^2^) for 10 s.

Following the application of each adhesive, a transparent acrylic cylinder (inner diameter: 3 mm; height: 4 mm) was fixed vertically on the flat dentin surface with a customized holding device. A nano-hybrid composite (Venus Diamond A3, Kulzer, Germany) was applied into the cylinder with a 2 mm increment and light-cured (BA Optima 10, B.A. international, UK, output > 800 mW/cm^2^) for 20 s at a 2 mm distance. For further processing, the acrylic cylinder was not removed.

All specimens were stored in demineralized water at room temperature for 24 h, 6 months, 12 months, or 24 months, respectively, before they were submitted to shear bond strength (SBS) testing. For each subgroup, n = 20 specimens were obtained.

### 2.3. Shear Bond Strength and Failure Mode Analysis

SBS was tested with a universal testing machine (Materialprüfmaschine 1446, Zwick GmbH & Co. KG, Ulm, Germany). Shear force was applied vertically to the bonding surface with a chisel-shaped loading device moving at a speed of 1 mm/min. Maximum load at debonding (*F*) was recorded and SBS was determined as *F*/*A* (*A* = bonding area of 7.07 mm^2^) (Software: testXpert V12.1, Zwick GmbH & Co. KG, Ulm, Germany).

**Figure 2:**
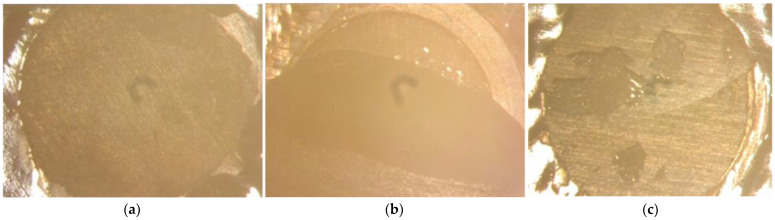
Exemplary microscopic images depicting the different failure modes: (**a**) adhesive failure directly at the interface between dentin and composite resin; (**b**) cohesive failure with at least 50% of the fracture surface within the dentin; (**c**) mixed failure.

Failure mode analysis was performed using a stereomicroscope (Stemi SV 11, Zeiss, Jena, Germany) at 16× magnification (Figure 2). Failures were classified as adhesive when the failure occurred at the interface between the dentin base and the nano-hybrid composite. Failures that occurred solely within dentin or within the nano-hybrid composite were considered cohesive. Failure mode was classified as mixed when both adhesive and cohesive failure occurred in one specimen.

### 2.4. Statistical Analysis

Statistical analyses were performed with the software SPSS Statistics for Macintosh (version 29.0.0.0, IBM, Armonk, NY, USA) and R (r-project version 4.3.1).

SBS data were tested for normal distribution using the Shapiro–Wilk test. As the majority of groups were distributed normally, parametric tests were applied. Separately, for the ER and SE adhesives, two-way analyses of variance (ANOVAs) were performed. Subsequently, one-way ANOVAs followed by Tukey post hoc tests (homogeneous variances) were performed to compare different groups at the same time point of aging or one group at different time points of aging.

Additionally, Weibull distribution parameters (Weibull modulus *m*, characteristic strength *σ*_0_) were calculated using the maximum likelihood estimation method at a 95% confidence level in MATLAB (version R2021a, 9.10.0.2015706, The Mathworks, Natick, MA, USA).

Separately, for the ER and SE adhesives, the effects of aging and MA-POSS-8 concentration on failure modes were assessed by χ^2^ tests. The overall level of significance was set at α = 0.05. *p*-values were adjusted for multiple testing according to Bonferroni–Holm.

## 3. Results

### 3.1. Shear Bond Strength

For the ER adhesive, both aging (*p* = 0.015) and MA-POSS-8 concentration (*p* < 0.001) had a significant effect on shear bond strength, while the interaction was not significant (*p* = 0.058). SBS did not change significantly over time in any group. However, after 12 months, SBS was significantly higher in the control group than for 10 wt.% MA-POSS-8 (*p*_adj._ = 0.006). After 24 months, 5 wt.% MA-POSS-8 led to significantly higher SBS than the control group (*p*_adj._ = 0.042) and 10 wt.% MA-POSS-8 (*p*_adj._ < 0.001) (Table 2).

The Weibull modulus ranged from 3.4 to 5.4 for all groups. The highest values were obtained within the control group and the lowest values with 10 wt.% MA-POSS-8. Characteristic strength decreased from 12 to 24 months for the control group but increased over time compared to 24 h for 5 wt.% MA-POSS-8. The 10 wt.% MA-POSS-8 reached the lowest values at all time points (Table 2).

For the universal adhesive applied in SE mode, both aging (*p* < 0.001) and MA-POSS-8 concentration (*p* = 0.039) had a significant effect on SBS; the interaction was not significant (*p* = 0.742). SBS in the control group and 5 wt.% MA-POSS-8 increased during aging compared to 24 h storage (*p*_adj._ ≤ 0.040). The universal adhesive modified with 10 wt.% MA-POSS 8 showed significantly higher bond strength after 12 months than after 24 h (*p*_adj._ = 0.003). At the different time points of aging, no significant differences between the groups were detected (Table 3).

The Weibull modulus varied from 4.6 to 7.6 for the MA-POSS-8 groups and from 4.3 to 10.2 for the control group. For all groups, the characteristic strength was lowest after 24 h of water storage and comparatively similar over all other storage periods (Table 3).

### 3.2. Failure Mode Analysis

For the ER adhesive, failure modes significantly varied by aging (*p*_adj._ < 0.001) and MA-POSS-8 concentration (*p*_adj._ = 0.016, Table 2). After 24 months, the control group and 10 wt.% MA-POSS-8 showed predominantly adhesive failures, whereas 5 wt.% MA-POSS-8 displayed considerably fewer adhesive and more cohesive failures.

For the universal adhesive applied in SE mode, neither aging nor MA-POSS-8 concentration impacted failure mode distribution significantly (Table 3). Overall, almost no adhesive but mostly cohesive failures were observed.

## 4. Discussion

Our null hypothesis that the dentin bond strength of the functionalized adhesives does not differ significantly from the dentin bond strength of the neat adhesives has to be partly rejected.

For the ER adhesive, the functionalization with 5 wt.% MA-POSS-8 led to a higher SBS after 24 months of water storage, while the incorporation with 10 wt.% MA-POSS-8 showed significantly lower bond strength values after 12 months, each compared to the control group. Nevertheless, the addition of either MA-POSS-8 concentration to the universal adhesive applied in SE mode obtained similar SBS values to the neat adhesive. Previous studies concerning the incorporation of POSS particles into dental adhesives indicated a good dispersion and interaction between both materials [4,6]. It was found that while multifunctional MA-POSS concentrations that exceeded 10 wt.% led to a deterioration of mechanical properties, lower concentrations either did not adversely affect mechanical qualities or were even able to enhance them [6,10,15,16].

Having these findings in mind, two different MA-POSS-8 concentrations were selected for our study. Our results indicate that whilst 5 wt.% MA-POSS-8 may improve the ER adhesive’s functionality over time, a concentration of 10 wt.% MA-POSS-8 might be overabundant as it decreased SBS compared to 5 wt.% MA-POSS-8 and the control group. For the SE adhesive, the MA-POSS-8 concentration showed no effect on SBS. For lower-concentrated MA-POSS-8, the fortified cross-linked structure of the incorporated adhesive leads to enhanced bonding. However, when applied in higher proportions, these effects seem to diminish. The reason may be the increased viscosity that results from higher MA-POSS-8 concentrations [6]. Higher viscosity leads to reduced wettability and thus less infiltrated dentin tubules, resulting in an inferior and less stable hybrid layer. For adequate bonding, ER adhesives rely on diffusion-based infiltration of the acid-etched exposed collagen fibril scaffold. SE adhesives are less dependent on diffusion processes because demineralization and penetration of the tooth surface proceed simultaneously [17]. This may be the reason why a potential increase in the viscosity through a higher MA-POSS-8 content showed no influence on the SBS of the SE adhesive while it decreased the SBS of the ER adhesive.

Many dental adhesives tend to obtain a relatively high immediate and short-term bond strength, while the clinical results are not always equally favorable. In vitro set-ups should therefore consider the long-term durability of the bonding, meaning an inclusion of aging procedures in the testing protocol [18]. Water storage is a frequently applied method to simulate aging processes in dental material testing [19,20]. A minimum storage period of 3 months is recommended to assess the possible water-induced degradation of the adhesive interface [21]. Hydrolysis leads to morphological changes in the collagen structures in dentin, and it alters macro- and microscopic composite structures and thus may decrease bond strength [19]. For the ER adhesive, all groups performed equally well at the first measurement after 24 h, whereas after the extended storage period of 24 months, the adhesive with 5 wt.% MA-POSS-8 presented significantly higher SBS than 10 wt.% MA-POSS-8 and the control group. Former investigations suggested MA-POSS-8 to increase the bond strength of dental adhesives by reason of its improved cross-link density, inducing a reduction in mesh size and interconnectivity of network pores, resulting in reduced water sorption [6]. Thus, the incorporation of POSS particles improves the hydrophilic stability of adhesives, leading to a higher aging resistance [4]. Furthermore, MA-POSS-8 is supposed to prevent bond strength degradation over time due to its mineralizing capacity [6,22]. Nonetheless, there are only a few studies that investigated the influence of aging on POSS particles [4,23]. As far as we know, at the present time, our investigation is the only one with extensively aged specimens.

Interestingly, the SBS of the universal adhesive applied in SE mode was lowest at the initial assessment after 24 h and significantly increased only afterwards, irrespective of the MA-POSS-8 concentration. An improvement in the initial bond strength after aging for Scotchbond Universal in SE mode was also observed in previous studies [24,25,26]. This phenomenon might be explained by post-cure polymerization that increases cross-linking density and thus strengthens the polymeric network [27]. Functional monomers incorporated into universal adhesives were found to decrease the adhesives’ degree of conversion, which was compensated by their concurrent ability to interact with the hydroxy-apatite [28] but may lead to a higher rate of post-cure polymerization. Furthermore, the universal adhesive contains 10-MDP, which creates a chemical bond with the calcium ions of dentin and thus provides a more stable bonding [29], maybe relativizing the impact of MA-POSS-8 on bonding performance.

When incorporated into different universal adhesives applied in SE mode, MA-POSS-8 did not show a significant influence on SBS [14], supporting the assumption that chemical bonding to the tooth structure overlies the beneficial effects of MA-POSS-8. Furthermore, the SE adhesives functionalized with MA-POSS-8 displayed only a slight increase in calcium phosphate precipitation compared to the respective neat adhesives [14]. Through its chemical bonding to calcium ions, 10-MDP possibly rivals the forming of calcium phosphate precipitates induced by MA-POSS-8.

The mineralizing capacity of MA-POSS-8 particles seems to rely on the hydrolysis of their Si-O bonds into Si-OH bonds that may then serve as starting points for mineral precipitation [11]. Exposed through an ER routine, dentinal collagen is highly vulnerable to hydrolytic degeneration due to possible nanoleakage from imperfect infiltration through the adhesive [17]. In a SE approach, the demineralization and infiltration of the tooth surface proceed simultaneously, enhancing the penetration of the adhesive and maintaining a hybrid layer less prone to hydrolysis [17]. The more stable hybrid layer of SE adhesives might lead to less available Si-OH bonds to induce mineral precipitation and may be an additional cause for the lack of influence of MA-POSS-8 on the SE adhesives’ bond strength.

Recently, the hybrid layer of an ER adhesive and a universal adhesive applied in SE mode doped with 10 wt.% MA-POSS-8 was analyzed after at least 13 weeks of storage in artificial saliva [30]. The presence of MA-POSS-8 in both adhesives leads to a predominant formation of amorphous calcium phosphate prenucleation clusters that are suggested to develop into hydroxyapatite during the crystallization process [31]. Electron microscopy images from the same investigation also showed that the storage duration significantly affected the MA-POSS-8 particles’ behavior, irrespective of the adhesive system: the specimens that were not stored in artificial saliva but immediately analyzed presented an MA-POSS-8 particle network that expanded across the entire adhesive layer as far as to the direct dentin–adhesive interface. After aging, the particle network seemed to dissolve beginning at the dentin–adhesive contact zone. The author assumed that the dendritic MA-POSS-8 network disintegrates from water fluctuation at the dentin–adhesive interface. Thus, particles are released that partly diffuse into the dentin’s collagen matrix to provide new ion agglomeration zones [30]. These findings support the assumption that MA-POSS-8 particles depend on hydrolytic processes to evolve their bioactive potential and consequently provide additional mechanical stabilization of the hybrid layer. Our results reflect that theory: MA-POSS-8 significantly improved the SBS of the adhesive only after 24 months of storage had elapsed. However, the investigation employed 10 wt.% MA-POSS-8, which in our study was not able to enhance bond strength for either adhesive. The increased mineralizing capacity of the higher MA-POSS-8 content might explain why mineral prenucleation clusters were observed equally for the ER and SE adhesives.

Despite the limitations of an in vitro experimental setup, there appears to be a correlation between bond strength results in vitro with bonding performance in vivo [21,32]. Bovine teeth show similar bonding behavior to human teeth irrespective of whether an ER or SE approach is selected [33,34]. Shear bond strength testing is one of the most commonly applied in vitro methods for bond strength analysis of dental materials [21,35]. However, its value is seen as ambiguous, since it tends to result in predominantly tensile stresses rather than shear stresses, non-uniform stress distribution, and stress concentration at the substrate area, reducing the validity of obtained results [36]. The advantage of SBS testing lies in its convenience as it is a relatively easy and efficient procedure [36]. Given that the priority of our investigation was to initially analyze the bond strength of adhesive systems incorporating MA-POSS-8 compared to the bonding performance of the neat adhesives, we determined SBS testing to be sufficient for our purposes. To gain a more detailed understanding of our bond strength data, Weibull statistics were applied. Since polymerized adhesives as well as resin composites and dentin are brittle materials, they hold a variability in strength-controlling flaws related to the specimen size [37]. Weibull statistics consider these consequences and thus enable conclusions on the reliability of bond strength data [37]. Low Weibull moduli reflect a poor reliability of the characteristic bond strength. Therefore, materials with higher Weibull moduli should be favored over those with lower values [37]. In addition to SBS data, we analyzed the failure mode distribution of our specimens. Once the SBS of the adhesive exceeds the stress limits of either dentin or composite resin, cohesive failures occur, which obviously only partly reflect interfacial stresses but nonetheless may be assumed to have stronger bonding, as the adhesive interface remains intact [37].

## 5. Conclusions

Keeping the limitations of an in vitro experiment in mind, we conclude that 5 wt.% MA-POSS-8 does not impair the bond strength of dental adhesives but might even be able to improve the long-term durability of adhesive systems. Under clinical conditions, the incorporation of MA-POSS-8 into adhesive systems might avail a prolonged longevity and aging resistance of adhesive restorations. Therefore, we encourage future studies on the clinical applicability of dental adhesives functionalized with MA-POSS-8.

## Figures and Tables

**Table 1:** LOT, manufacturer, and composition of the adhesive systems of Scotchbond Universal (universal adhesive applied in SE mode) and Solobond Plus (ER adhesive) as provided by the manufacturers.

Adhesive	LOT	Manufacturer	Components
Scotchbond Universal	902198 905248	3M, St. Paul, MN, USA	15–25 wt.% 2-hydroxyethyl methacrylate; 15–25 wt.% bisphenol A diglycidyl ether dimethacrylate 10–20 wt.% 2-propenoic acid, 2-methyl-, reaction products with 1,10-decanediol and phosphorus oxide 10–15 wt.% ethanol 10–15 wt.% water 7–13 wt.% 2-propenoic acid, 2-methyl-,3- (trimethoxysilyl)propyl ester, reaction products with vitreous silica 1–5 wt.% copolymer of acrylic and itaconic acid <2 wt.% camphorquinone <2 wt.% dimethylaminobenzoat(-4) <1 wt.% (dimethylamino)ethyl methacrylate
Solobond Plus	Primer: 1913352 2117451 Bonding: 1915318 2122279	VOCO GmbH, Cuxhaven, Germany	Primer: 10–25 wt.% 2-hyydroxyethyl methacrylate 10–25 wt.% acetone 10–25 wt.% hydroxypropylmethacrylate ≤2.5 wt.% catalyst Bonding: 10–25 wt.% bisphenol A-glycidyl methacrylate 10–25 wt.% triethylene glycol dimethacrylate 5–10 wt.% 2-hydroxyethyl methacrylate ≤2.5 wt.% catalyst

**Table 2:** SBS (shear bond strength, mean ± standard derivation [SD]), Weibull parameters (characteristic strength *σ*_0_, Weibull modulus *m* with 95% confidence intervals [CIs]), and failure modes of the ER adhesive Solobond Plus depending on the MA-POSS-8 concentration and storage period. Significant differences within one column are shown by different lowercase superscript letters; different uppercase superscript letters indicate significant differences within one row.

Parameter	POSS (wt.%)	24 h Aging	6 Months Aging	12 Months Aging	24 Months Aging
SBS MPa (mean ± SD)	0	17.4 ± 4.9 ^aA^	18.2 ± 4.1 ^aA^	18.7 ± 5.2 ^bA^	14.6 ± 3.6 ^aA^
	5	14.3 ± 4.3 ^aA^	18.3 ± 4.8 ^aA^	17.3 ± 4.9 ^abA^	17.9 ± 5.1 ^bA^
	10	13.7 ± 4.4 ^aA^	15.1 ± 4.9 ^aA^	14.3 ± 3.0 ^aA^	12.8 ± 4.1 ^aA^
*σ*_0_ MPa [95% CI]	0	19.2 [17.2–21.5]	19.8 [18.2–21.6]	20.7 [18.5–23.1]	15.9 [14.4–17.6]
	5	15.9 [14.1–17.9]	20.2 [18.0–22.6]	19.2 [16.9–21.8]	19.8 [17.5–22.4]
	10	15.3 [13.3–17.5]	16.8 [14.6–19.2]	15.5 [14.1–16.9]	14.2 [12.5–16.1]
*m* [95% CI]	0	4.1 [2.9–5.9]	5.4 [3.8–7.6]	4.1 [2.9–5.8]	4.6 [3.3–6.4]
	5	3.8 [2.7–5.4]	4.0 [2.9–5.5]	3.7 [2.7–5.0]	3.8 [2.7–5.2]
	10	3.4 [2.5–4.7]	3.4 [2.4–4.7]	5.1 [3.7–7.0]	3.7 [2.6–5.2]
Failure mode % (adhesive; cohesive; mixed)	0	25; 35; 40	20; 30; 50	15; 45; 40	65; 10; 25
	5	15; 10; 75	20; 20; 60	15; 30; 55	25; 50; 25
	10	10; 15; 75	25; 25; 50	10; 5; 85	70; 5; 25

**Table 3:** SBS (shear bond strength, mean ± standard derivation [SD]), Weibull parameters (characteristic strength *σ*_0_, Weibull modulus *m* with 95% confidence intervals [CIs]), and failure modes of the universal adhesive Scotchbond Universal applied in SE mode depending on the MA-POSS-8 concentration and storage period. Significant differences within one column are shown by different lowercase superscript letters; different uppercase superscript letters indicate significant differences within one row.

Parameter	POSS (wt.%)	24 h Aging	6 Months Aging	12 Months Aging	24 Months Aging
SBS MPa (mean ± SD)	0	19.1 ± 5.2 ^aA^	25.8 ± 4.4 ^aB^	25.1 ± 3.0 ^aB^	23.6 ± 4.6 ^aB^
	5	20.0 ± 4.6 ^aA^	24.2 ± 5.4 ^aB^	24.8 ± 4.4 ^aB^	23.9 ± 3.8 ^aB^
	10	18.6 ± 4.1 ^aA^	22.2 ± 4.8 ^aAB^	24.2 ± 4.1 ^aB^	21.8 ± 6.3 ^aAB^
*σ*_0_ MPa [95% CI]	0	21.0 [18.8–23.3]	27.7 [25.8–29.7]	26.4 [25.2–27.6]	25.5 [23.7–27.4]
	5	21.7 [20.0–23.5]	26.2 [24.1–28.6]	26.7 [24.8–28.7]	25.4 [23.9–27.0]
	10	20.2 [18.5–22.0]	24.0 [22.0–26.1]	25.9 [24.3–27.7]	23.8 [21.6–26.3]
*m* [95% CI]	0	4.3 [3.1–6.1]	6.6 [4.7–9.2]	10.2 [7.2–14.6]	6.2 [4.4–8.9]
	5	5.8 [3.9–8.4]	5.4 [3.8–7.7]	6.3 [4.6–8.8]	7.6 [5.4–10.5]
	10	5.3 [3.8–7.4]	5.5 [3.9–7.6]	7.0 [4.9–9.8]	4.6 [3.1–6.8]
Failure mode % (adhesive; cohesive; mixed)	0	5; 75; 20	0; 70; 30	0; 75; 25	0; 70; 30
	5	0; 85; 15	0; 80; 20	0; 55; 45	0; 85; 15
	10	0; 90; 10	0; 80; 20	0; 90; 10	0; 70; 30

## Data Availability

The data presented in this study are available on request from the corresponding author.
